# Impact of preoperative handgrip strength and frailty on early postoperative loss of independence in patients with colorectal cancer

**DOI:** 10.3389/fonc.2026.1878444

**Published:** 2026-07-01

**Authors:** Dongqin Zhao, Fan He, Hongjiang Liu, Xiaoqing Qing

**Affiliations:** 1 Department of Nursing, The First Affiliated Hospital of Chongqing Medical University, Chongqing, China; 2 Department of Gastrointestinal Surgery, The First Affiliated Hospital of Chongqing Medical University, Chongqing, China; 3 Department of Radiology, The First Affiliated Hospital of Chongqing Medical University, Chongqing, China; 4 Department of Anesthesiology, The First Affiliated Hospital of Chongqing Medical University, Chongqing, China

**Keywords:** colorectal cancer, frailty, handgrip strength, loss of independence, preoperative depression

## Abstract

**Objective:**

This study aimed to identify factors associated with early postoperative loss of independence (LOI) and assess the utility of preoperative handgrip strength (HGS) and frailty evaluation among patients undergoing colorectal cancer (CRC) resection.

**Methods:**

We prospectively evaluated HGS, frailty, and depression among patients with CRC enrolled at our center from February 2024 to February 2025. The associations between HGS and perioperative outcomes were analyzed. Subsequently, a predictive model for postoperative LOI was developed based on the above three variables.

**Results:**

A total of 258 patients with CRC were enrolled, and 60 (23.3%) developed early postoperative LOI. Patients with preoperative low HGS had shorter operative time (*p* = 0.020) and increased postoperative complications (*p* = 0.002), particularly pneumonia (*p* = 0.031). Delayed first defecation (*p* = 0.008), prolonged time to full oral nutrition (*p* = 0.024), and extended length of stay (*p* = 0.002) were also observed in these patients. Multivariate analysis showed that American Society of Anesthesiologists (ASA) score ≥3 [odds ratio (OR) = 2.556, *p* = 0.019], preoperative moderate frailty (OR = 9.504, *p* = 0.002), and low HGS (OR = 2.452, *p* = 0.01) were adverse predictors of LOI. The receiver operating characteristic (ROC) curve revealed a moderate predictive performance of the model, with an area under the curve (AUC) of 0.740 [95% confidence interval (CI): 0.657–0.823].

**Conclusion:**

Patients with low HGS are prone to increased postoperative complications and delayed postoperative recovery. Postoperative LOI is independently correlated with ASA score ≥ 3, low HGS, and moderate frailty. Therefore, assessment of frailty and HGS prior to radical resection of CRC facilitates preoperative risk stratification.

## Introduction

Colorectal cancer (CRC) ranks among the most prevalent primary malignant tumors of the digestive tract (1). While chemotherapy, targeted therapy, and immunotherapy have been widely applied in clinical practice, surgery is still the main curative approach for CRC (2). Surgery is only the cornerstone of treatment, and maintaining long-term physical function and postoperative independence represents the top priority for patients with CRC (3). Nevertheless, radical resection for CRC, as a major abdominal surgery, carries an elevated risk of postoperative complications and postoperative loss of independence (LOI).

At present, preoperative risk stratification prior to CRC radical resection is still challenging, largely due to the reliance on conventional, costly auxiliary tests such as cardiopulmonary function and laboratory examinations (4). Some elderly patients have severe frailty, which makes surgeons hesitant to proceed with surgery, while others maintain good physical activity. Accumulated evidence suggests that age is not an independent predictor of adverse postoperative events among patients with CRC (5). Hence, a simple predictive tool is urgently required to estimate short-term postoperative outcomes and LOI following colorectal surgery.

Handgrip strength (HGS) is a simple, readily available preoperative measure that can be assessed objectively and accurately. It correlates with patients’ nutritional status, physical fitness, aging, and immune function (6). As a direct reflection of muscle strength, HGS plays a vital role in the diagnosis of sarcopenia (7). Sarcopenia raises the risk of mobility impairment, prolonged length of stay (LOS) and loss of basic self-care ability, all of which are detrimental to the preservation of postoperative independence.

Frailty reflects diminished physiological reserve and stress resilience, and is an established independent predictor of adverse postoperative events such as major complications, prolonged LOS, and unplanned readmission (8). An increasing number of surgeons have recognized the importance of frailty for preoperative risk stratification. In line with this, the American College of Surgeons and the American Geriatrics Society have issued joint guidelines to recommend routine frailty assessment among elderly surgical patients (9). The Edmonton Frail Scale (EFS), a brief tool for evaluating preoperative frailty, takes approximately 5 min to complete and does not require professional geriatric expertise for administration (10). It includes assessments of cognitive and psychosocial status, and its validity for frailty evaluation has been fully confirmed.

In addition, growing evidence suggests that depression is associated with multiple adverse postoperative outcomes (11). Lee et al. (12) reported that preoperative anxiety and depression negatively affect postoperative quality of life (QoL) after hepatocellular carcinoma resection. The Patient Health Questionnaire-9 (PHQ-9) has been well validated for depression screening in previous studies (13). Although the PHQ-9 contains fewer items than most conventional depression scales, it yields comparable sensitivity and specificity. The scale consists of nine items based on the diagnostic criteria of the *Diagnostic and Statistical Manual of Mental Disorders, Fourth Edition*.

Previous studies (14, 15) have evaluated the roles of HGS, frailty, and preoperative depression in poor postoperative outcomes following abdominal surgery for gastric cancer (GC), biliary tract cancer, and CRC. However, most previous studies have mainly focused on perioperative safety indicators, such as postoperative delirium, perioperative mortality, postoperative LOS, and overall survival. With the improvement of medical care quality, greater emphasis has been placed on not only surgical safety and long-term survival but also postoperative QoL and the preservation of independent living ability. Therefore, we aimed to assess the risk of postoperative LOI via preoperative evaluation of HGS, frailty, and depression in patients undergoing radical resection of CRC.

In this study, we investigated the risk factors for early postoperative LOI in patients with CRC and further explored the predictive value of preoperative HGS and frailty assessment.

## Methods

### Study participants

This was a prospective observational study. We prospectively enrolled patients diagnosed with CRC who were admitted to the Department of Gastrointestinal Surgery, the First Affiliated Hospital of Chongqing Medical University, from February 2024 to February 2025, and preoperatively assessed their depression status, frailty level, and HGS.

Inclusion criteria: (1) Patients with a pathological diagnosis of colorectal adenocarcinoma; and (2) age ≥ 18 years old.

Exclusion criteria: (1) Patients who required preoperative chemotherapy or declined surgical treatment; (2) patients who underwent palliative ostomy due to intestinal obstruction; (3) patients who received endoscopic tumor resection; and (4) patients with preoperative LOI.

This study was approved by the Ethics Committee of the First Affiliated Hospital of Chongqing Medical University (Approval ID: K2024-020-01). All participants provided written informed consent for the prospective use of their anonymized clinical data for health-related research.

### Data collection

Preoperative depression, frailty, and HGS were prospectively evaluated in all patients with CRC who presented to our center or received radical resection for CRC after neoadjuvant therapy. Preoperative HGS (in kilograms) was measured on admission using a Takei TKK 5401 digital dynamometer. Patients stood upright with both arms hanging naturally, and two HGS readings were obtained from the dominant hand with adequate rest in between. The higher value was documented as the final HGS. In accordance with the Asian criteria (16), the HGS cutoff values were 28 kg for men and 18 kg for women. Readings below the thresholds were defined as low HGS, while those above were defined as high HGS.

Following HGS measurement, nurses guided patients to finish the EFS and PHQ-9 questionnaires, whose validity was checked right after completion. Prior to enrollment, all patients were fully informed of the objectives of this survey and the confidentiality of personal information. Questionnaires were administered only after participants provided written informed consent. The EFS and PHQ-9 have been well validated in previous studies (17). The EFS was applied to evaluate preoperative frailty in surgical patients. This scale consists of 11 items, 9 of which are self-reported. Its total score ranges from 0 to 17, with stratification as follows: 0–3 for no frailty, 4–5 for minor frailty, 6–8 for moderate frailty, and 9–17 for major Low anterior resection syndrome (**Supplementary Table 1**). The PHQ-9 comprises nine items and serves dual functions: diagnosing depression and evaluating the severity of depressive symptoms. Its total score ranges from 0 to 27. The severity classifications are as follows: 0–4 (no depression), 5–9 (minor depression), 10–14 (moderate depression), 15–19 (moderate-major depression), and 20–27 (major depression) (**Supplementary Table 2**).

In addition, five modified frailty indices (mFIs) were adopted to evaluate the risk of adverse health outcomes (18). As a well-validated frailty metric, the mFI is calculated based on four comorbid conditions and one functional status variable: chronic obstructive pulmonary disease, congestive heart failure within 30 days prior to surgery, diabetes treated with oral agents or insulin, hypertension requiring pharmacotherapy, and preoperative functional status (independent, partially independent, and dependent). One point is assigned for each positive item, yielding a total possible score of 5. A score of 2 or higher is defined as frailty.

We prospectively extracted the following data of patients with CRC via the electronic medical record system: age, gender, body mass index (BMI), smoking history, alcohol consumption, comorbidities, American Society of Anesthesiologists (ASA) score, primary tumor location, neoadjuvant chemotherapy, tumor–node–metastasis (TNM) stage, preoperative hemoglobin (Hb), preoperative albumin (Alb), operative duration, intraoperative blood loss, time to first defecation, time to full oral diet, duration of antibiotic administration, indwelling time of abdominal drainage tube, postoperative complications, LOS, reoperation, hospital readmission, and in-hospital mortality.

The level of independence was defined as the change in the level of activities of daily living (ADL) from before surgery to the 14th day after discharge (ADL scale, classified as independent, partially dependent, and dependent). The Barthel Index (BI) was used by attending nurses to assess patients’ independence at admission, and prior training was provided to all relevant nursing staff to maintain assessment reliability. Follow-up assessments and treatment arrangement were conducted 14 days post-discharge, with the BI applied again to measure functional independence (19). Using established clinical grading standards, participants were classified as independent (BI ≥60), partially dependent (BI 41–59), or dependent (BI ≤40). Early postoperative LOI, defined as partial or complete dependence (BI ≤59), was selected as the primary endpoint (20).

The severity of complications is graded according to the Clavien–Dindo (CD) classification (21), where CD grade 1 or 2 complications are considered mild and CD grade 3 or higher complications are considered serious.

Reoperation is defined as an unplanned surgery within postoperative 30 days. Readmission is defined as an unplanned hospitalization within 30 days of discharge. Mortality was defined as patients who died within 30 days of initial surgery.

### Statistical analysis

SPSS 27.0 (IBM Corp, Armonk, NY, USA) was used for statistical analysis, and GraphPad Prism 9.0 was applied to plot receiver operating characteristic (ROC) curves. Normally distributed continuous variables were presented as mean ± standard deviation, and between-group differences were compared using the Student’s *t*-test. Non-normally distributed continuous variables were described as median and interquartile range, with the Mann–Whitney *U* test adopted for group comparisons. Categorical variables were expressed as frequencies and percentages, and differences were assessed via the chi-square test or Fisher’s exact test. Univariate and multivariate logistic regression analyses were performed to identify independent risk factors for LOI following radical CRC resection, and results were presented as odds ratios (ORs) with 95% confidence intervals (CIs). A *p*-value < 0.05 was considered statistically significant.

Independent predictors derived from multivariate regression were incorporated to establish a prediction model, and the corresponding ROC curve was plotted. Model performance was assessed by the area under the curve (AUC). To prevent overfitting, the multivariable logistic regression model underwent internal validation via 1,000-bootstrap resampling. The Hosmer–Lemeshow test and calibration curves were used to evaluate model calibration. The optimal cutoff value was determined based on the maximum Youden index, followed by calculations of sensitivity, specificity, positive predictive value (PPV), and negative predictive value (NPV). Decision curve analysis (DCA) was performed to assess the clinical net benefit of the prediction model across different threshold probabilities.

## Results

**Figure 1:**
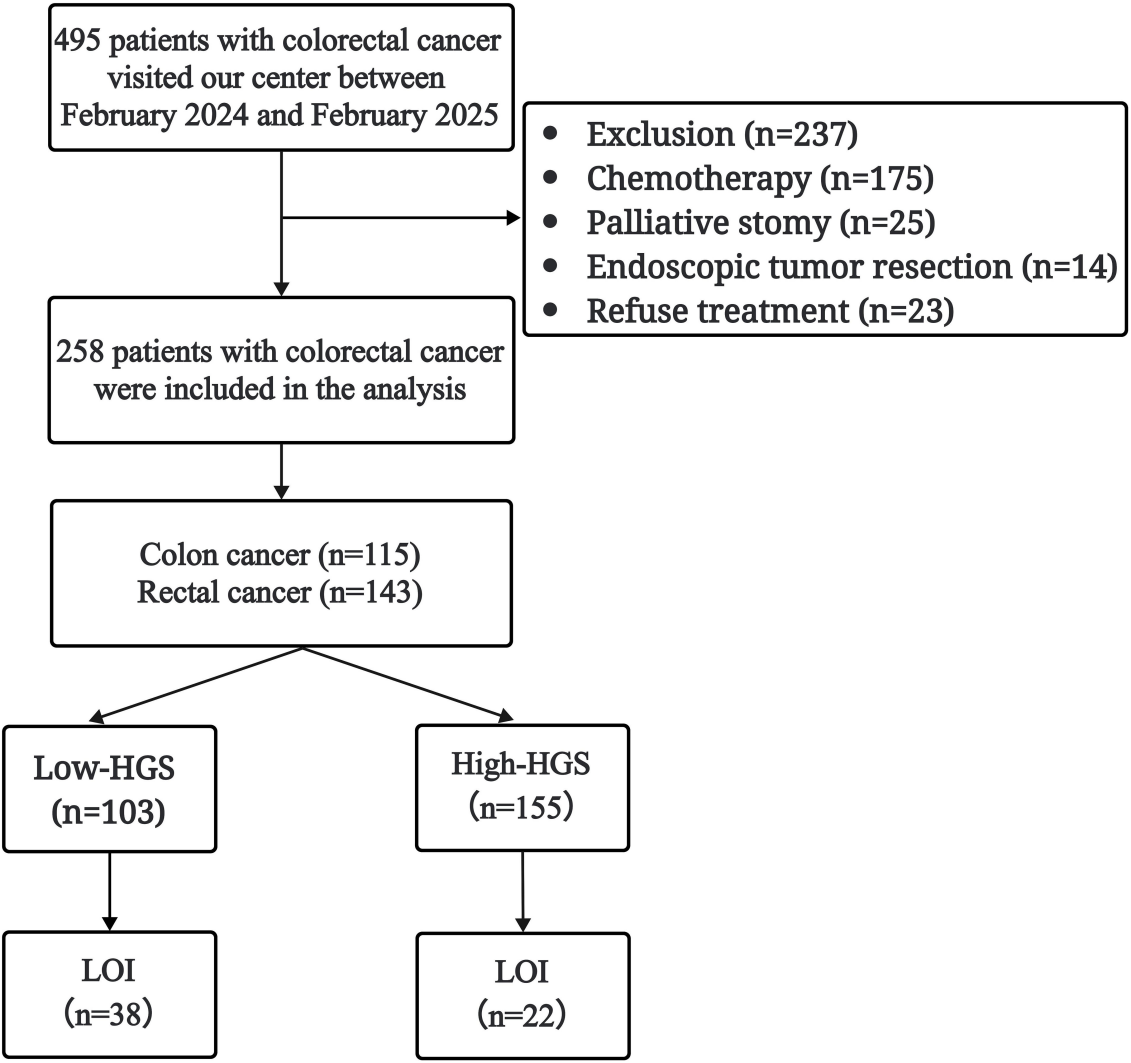
Flowchart of patient enrollment.

A total of 495 patients with CRC who were initially visited in our center or scheduled for radical resection of CRC after neoadjuvant therapy admitted to the Department of Gastrointestinal Surgery, First Affiliated Hospital of Chongqing Medical University from February 2024 to February 2025. Excluded the 175 patients received chemotherapy, 25 patients underwent palliative ostomy, 14 patients received endoscopic tumor resection and 23 patients refused received any treatment, 258 patients included eventually analysis, involving 113 patients with colon cancer and 143 patients with rectal cancer, in which 60 (23.3%) patients developed postoperative early LOI (**Figure 1**). The demographics, tumor characteristics, and preoperative frailty status of patients included in the study are shown in **Table 1**, and the detailed EFS scores and HGS values of the patients are shown in **Supplementary Figure 1**.

**Table 1:** Demographic and tumor characteristics of patients with CRC.

Variables	n(%)/Mean±SD
Age (years)	63.18±11.39
Sex (male)	137 (53.1%)
BMI (kg/m^2^)	23.29±3.47
Smoking	71 (27.5%)
Alcohol consumption	53 (20.5%)
Hypertension	90 (34.9%)
Diabetes	41 (15.9%)
Chronic pulmonary disease	6 (2.3%)
Coronary artery disease	17 (6.6%)
Type of cancer	
Colon cancer	115 (44.6%)
Rectal cancer	143 (55.4%)
TNM stage	
I	49 (19.0%)
II	105 (40.7%)
III	85 (32.9%)
IV	19 (7.4%)
Neoadjuvant chemotherapy	43 (16.7%)
ASA score	
1	20 (7.8%)
2	117 (45.3%)
3	121 (46.9%)
preoperative hemoglobin (g/L)	119.96±22.01
Preoperative albumin (g/L)	39.39±4.87
Depression	
No	232 (89.9%)
Minor	24 (9.3%)
Moderate	2 (0.8%)
Frailty	
No	209 (81.0%)
Minor	32 (12.4%)
Moderate	17 (6.6%)
Handgrip strength (kg)	
Male	28.34±6.97
Female	19.05±6.06
Modified frailty index (≥2)	47 (18.2%)
Barthel index score	86.1±8.3

CRC, colorectal cancer; SD, standard deviation; BMI, body mass index; TNM, tumor-node-metastasis; ASA, American Society of Anesthesiologists.

Compared with patients with high HGS, those with low HGS had shorter operative time (*p* = 0.020) and a higher incidence of postoperative complications (*p* = 0.002), particularly pneumonia (*p* = 0.031). Patients in the low-HGS group also presented with longer time to first defecation (*p* = 0.008), longer time to full oral nutrition (*p* = 0.024), prolonged antibiotic administration (*p* = 0.033), longer indwelling time of abdominal drainage tube (*p* = 0.008), and extended LOS (*p* = 0.002). No significant intergroup differences were observed in intraoperative blood loss (*p* = 0.063), postoperative anastomotic leakage (*p* = 0.272), or intra-abdominal infection (*p* = 0.993). Likewise, the rates of postoperative reoperation (*p* = 0.391), readmission (*p* = 0.278), and mortality (*p* = 0.399) were comparable between the two groups (**Table 2**).

**Table 2:** Comparison of perioperative outcomes between two groups.

	Low-HGS	High-HGS	
Variables	(n=103) %	(n=155) %	p
Operative time (min)	125 (100-150)	135 (110-175)	0.020*
Intraoperative blood loss (ml)	20 (20-50)	50 (20-50)	0.063
Overall complications	33(32.0%)	25(16.1%)	0.002*
Clavien-Dindo grade			0.056
I	5 (4.9%)	3 (1.9%)	
II	22 (21.4%)	18 (11.6%)	
IIIa	3 (2.9%)	2 (1.3%)	
IIIb	3 (2.9%)	2 (1.3%)	
Pneumonia	11(10.7%)	6(3.9%)	0.031*
Anastomotic leakage	5(4.9%)	3(1.9%)	0.272
Intra-abdominal infection	12(11.7%)	18(11.6%)	0.993
Time of abdominal drainage tube (days)	7 (6-9)	7 (6-8)	0.008*
Time of first defecation (days)	4 (3-5)	3 (3-4)	0.008*
Full oral nutrition time (days)	6 (5-8)	5 (4-7)	0.024*
Duration of antibiotic use (days)	2 (2-7)	2 (2-4)	0.033*
Postoperative hospital stay (days)	8 (7-11)	8 (6-10)	0.002*
Barthel index score			<0.001*
≥60	65	133	
40∼60	25	17	
<40	13	5	
Reoperation	3(2.9%)	2(1.3%)	0.391
Mortality	1(1.0%)	0(0.0%)	0.399
Readmission	0(0.0%)	3(1.9%)	0.278

HGS, handgrip strength; *p<0.05.

Univariate analysis of risk factors for early postoperative LOI revealed that age ≥ 80 years (*p* < 0.001), BMI < 24 (*p* = 0.017), ASA score ≥ 3 (*p* < 0.001), preoperative moderate frailty (*p* < 0.001), low HGS (*p* < 0.001), mFI ≥ 2 (*p* = 0.023), preoperative Hb < 100 (*p* < 0.001), and preoperative Alb < 37 (*p* < 0.001) were significantly associated with early postoperative LOI. Subsequent multivariate regression analysis incorporating the above variables demonstrated that ASA score ≥ 3 (OR = 2.556, 95% CI: 1.171–5.577, *p* = 0.019), preoperative moderate frailty (OR = 2.504, 95% CI: 1.403–4.469, *p* = 0.002), and low HGS (OR = 2.452, 95% CI: 1.242–4.843, *p* = 0.010) were independent risk factors for early postoperative LOI (**Table 3**). No significant correlation was observed between preoperative depression and LOI among patients who received CRC surgery.

**Table 3:** Univarate and multivariate analysis of risk factors for the postoperative LOI.

	Univarate		Multivariate	
Variables	OR(95% CI)	p	OR(95% CI)	p
Age (≥80 vs <80) (years)	11.412 (2.981~43.693)	<0.001	2.167 (0.554~8.483)	0.136
Sex (male vs female)	1.176 (0.660~2.096)	0.583		
BMI (<24 vs ≥24) (kg/m^2^)	2.247 (1.158~4.357)	0.017	1.241 (0.572~2.693)	0.602
Smoking (yes vs no)	1.301 (0.693~2.441)	0.412		
Alcohol consumption (yes vs no)	0.834 (0.399~1.743)	0.629		
Hypertension (yes vs no)	1.333 (0.735~2.417)	0.343		
Diabetes (yes vs no)	0.915 (0.410~2.044)	0.829		
Chronic pulmonary disease (yes vs no)	3.421 (0.672~17.413)	0.139		
Coronary artery disease (yes vs no)	1.409 (0.476~4.173)	0.536		
ASA score (3 vs 1/2)	3.891 (2.073~7.305)	<0.001	2.556 (1.171~5.577)	0.019*
Cancer type (rectal cancer vs colon cancer)	0.875 (0.490~1.564)	0.653		
Metastasis (yes vs no)	0.884 (0.833~2.775)	0.833		
NCT (yes vs no)	0.594 (0.250~1.414)	0.240		
Depression		0.245		
Minor vs no	2.129 (0.881~5.149)	0.093		
Moderate vs no	0.000 (0.000~0.000)	0.999		
Frailty		<0.001		0.002*
Minor vs no	2.113 (0.924~4.834)	0.076		
Moderate vs no	15.108 (4.663~48.951)	<0.001	2.504 (1.403~4.469)	
Handgrip strength (low vs high)	3.354 (1.934~6.460)	<0.001	2.452 (1.242~4.843)	0.010*
Modified frailty index (≥2 vs <2)	2.214 (1.118~4.382)	0.023	1.349 (0.498~3.651)	0.532
Preoperative Hb (<100 vs ≥100) (g/L)	3.589 (1.805~7.138)	<0.001	2.163 (0.943~4.961)	0.058
Preoperative Alb (<37 vs ≥37) (g/L)	3.283 (1.742~6.186)	<0.001	1.510 (0.694~3.284)	0.296

LOS, loss of independence; OR, Odds Ratio; CI, Confidence Interval; BMI, bosy mass index; ASA, American Society of Anesthesiologists; NCT, Neoadjuvant Chemotherapy; Hb, Hemoglobin; Alb, albumin; *p<0.05.

**Figure 2:**
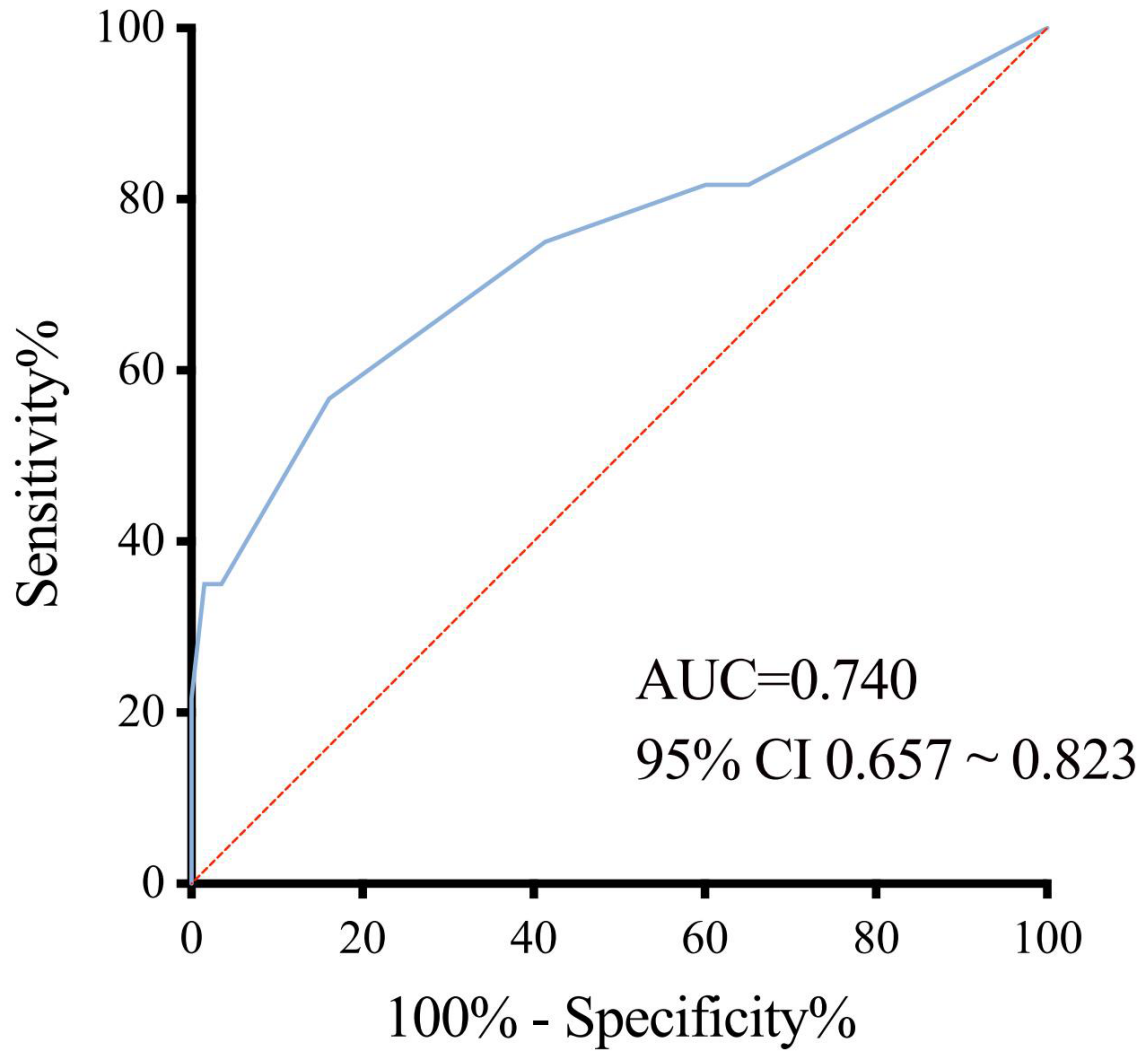
ROC curve for the prediction model of postoperative LOI (AUC, area under the curve).

**Figure 3:**
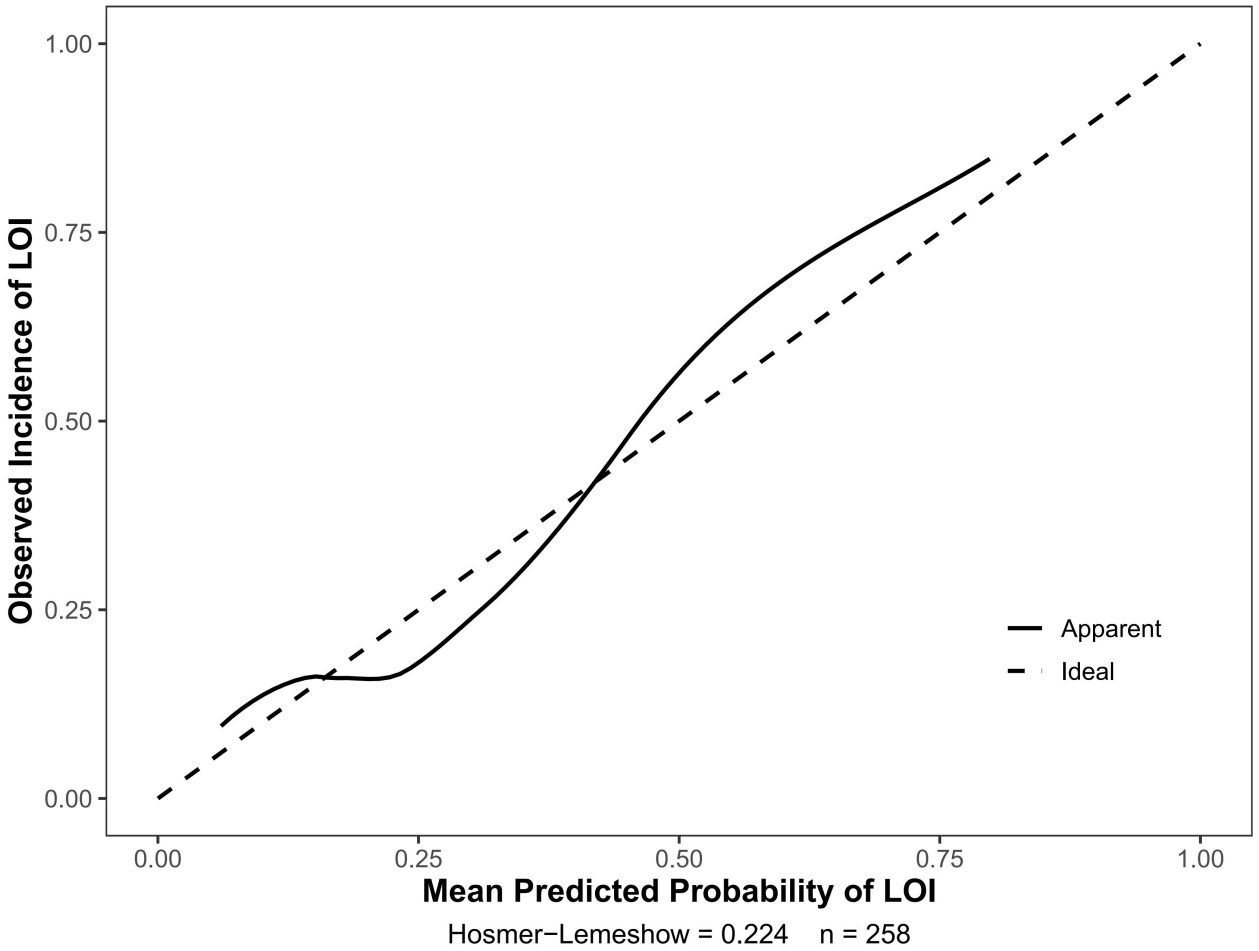
Calibration curve for the prediction model of postoperative LOI.

Furthermore, we developed a predictive model based on preoperative ASA score, frailty, and HGS to early predict postoperative LOI in patients with CRC. In multivariate analysis, the Hosmer–Lemeshow goodness-of-fit test yielded a value of 0.224, indicating no apparent overfitting of the model. ROC curve analysis showed an AUC of 0.740 (95% CI: 0.657–0.823), suggesting the moderate predictive performance of the model. At the optimal cutoff value determined by the maximum Youden index, the model exhibited a sensitivity of 70.0%, a specificity of 75.8%, a PPV of 46.7%, and an NPV of 84.3%. The AUC curve and calibration curve are shown in **Figures 2**, **3**, respectively. In addition, the DCA curve showed that the model provided higher net benefit than both “treat-all” and “treat-none” strategies over a wide range of thresholds, indicating favorable clinical utility (**Supplementary Figure 2**).

## Discussion

A rapid return to daily life is a universal expectation among patients undergoing radical resection for CRC. Postoperative LOI represents a major barrier to their postoperative rehabilitation. Early identification of patients at high risk of LOI and intensified perioperative management are critical to addressing this clinical issue. The present study demonstrated that patients with preoperative low HGS tended to experience more postoperative complications, particularly pneumonia, alongside delayed postoperative recovery. In addition, we constructed a simple and practical prediction tool for early postoperative LOI based on multivariate analysis. The model incorporating preoperative ASA score ≥ 3, moderate frailty, and low HGS performed moderately well in identifying patients at high risk of LOI. Accordingly, preoperative assessment of frailty and HGS in patients with CRC undergoing radical resection can aid risk stratification. Targeted pre-rehabilitation and optimized perioperative care for high-risk individuals may help prevent the development of LOI.

Previous studies (22–24) have verified that preoperative low HGS is an adverse factor affecting short-term postoperative outcomes in patients with upper gastrointestinal malignancies following radical resection. Kurita et al. (23) reported that low HGS independently predicted postoperative pneumonia after thoracoscopic–laparoscopic esophagectomy for esophageal cancer. Matsui et al. (24) demonstrated that preoperative low HGS defined by Asian criteria was correlated with infectious complications after gastrectomy. Consistent with these findings, our study showed that patients with low preoperative HGS had a higher overall rate of postoperative complications, particularly pneumonia. Preoperative HGS is closely linked to age, comorbidities, nutritional status, and muscle mass, and can objectively reflect patients’ physical condition (25). Additionally, low HGS is strongly associated with frailty (26). To date, no universal cutoff value for HGS has been established. In the present study, we adopted the Asian criteria to define HGS thresholds. While some researchers (27) set cutoff values based on the lowest 20% of HGS distribution stratified by sex, our results indicate that the cutoffs from Asian criteria serve as effective predictors of postoperative complications and prolonged recovery.

HGS is determined by overall muscle mass (21). Patients with low HGS exhibit reduced limb muscle strength, which impairs ambulation. Under the concept of enhanced recovery after surgery, early mobilization is routinely recommended by surgeons. Nevertheless, patients with low HGS often struggle to comply with early ambulation protocols. This leads to prolonged bed rest, delayed intestinal function recovery, and an elevated risk of pneumonia. Furthermore, HGS is strongly correlated with nutritional status (28). Malnourished patients typically experience slow postoperative recovery and are susceptible to hypoproteinemia, which further increases the incidence of infectious complications and prolongs LOS. Additionally, poor nutritional status is accompanied by decreased visceral fat. This may account for the shorter operative time observed in patients with low HGS in the present study.

Radical resection is the primary curative treatment for CRC. The clinical benefits of surgery can be fully achieved only if patients preserve preoperative activity and self-care abilities and resume social life in a timely manner. This study revealed that patients with preoperative ASA score ≥3, moderate frailty, and low HGS face a higher risk of LOI following major CRC surgery, even when the malignancy is surgically eradicated. Hence, comprehensive preoperative assessment is essential for optimizing treatment strategies. Most previous studies (8, 29) focused on frailty evaluation merely among elderly patients. With the general improvement in physical fitness among older adults, age is no longer an independent predictor of adverse postoperative events after major surgery. By contrast, a growing number of younger patients have diminished physical resilience. Insufficient daily physical activity makes them vulnerable to psychological disturbances, depression, and reduced HGS. Accordingly, the present study enrolled all patients with CRC aged over 18 years. Preoperative HGS, depression, and frailty were systematically assessed to explore their associations with short-term adverse outcomes and LOI.

Patients with an ASA score ≥ 3 in this study had a higher risk of developing early postoperative LOI. A prior study (30) demonstrated that an ASA score ≥ 3 acts as an independent risk factor for complications following major abdominal surgery. This scoring system assesses anesthesia-related risk and reflects patients’ general physical status and tolerance to surgical stress. Favorable postoperative recovery is critical for minimizing the occurrence of LOI. Sakurai et al. (8) reported that major postoperative complications (CD ≥ 3) were an independent predictor of LOI in patients undergoing major abdominal surgery.

In this study, patients with moderate frailty had a 2.5-fold higher risk of LOI than non-frail patients, while mild frailty was not associated with a significant difference in LOI risk. An EFS score greater than 6 was identified as an independent predictor of early postoperative LOI. Montroni et al. (31) similarly demonstrated that frailty negatively affects QoL and functional recovery among patients with CRC. Berian et al. (32) found that LOI is closely linked to preoperative cognitive dysfunction and postoperative delirium. Because of the lack of reliable tools for identifying postoperative delirium in our cohort, we did not analyze whether this condition acts as an independent predictor of early postoperative LOI. Furthermore, no significant association was found between preoperative depression and postoperative LOI. Considering the sustained impact of psychological status, we will perform long-term follow-up to explore how preoperative depression influences long-term QoL and oncological outcomes, and report these results in future studies.

This study has several limitations. First, it was a single-center study with a relatively modest sample size of patients with CRC. Second, the HGS cutoff values were determined based on Asian criteria. While these criteria effectively predict poor short-term prognosis among patients with CRC with low HGS, their generalizability to Western populations requires further validation. Third, early postoperative LOI was defined according to patients’ ability to perform ADL at 14 days after discharge, and long-term LOI was not assessed. We are currently conducting another prospective study to explore the association between chemotherapy and LOI, and will continue long-term follow-up to monitor patients’ daily functional status after surgery.

## Conclusion

Approximately one in four patients undergoing radical resection for CRC developed early postoperative LOI. Patients with low preoperative HGS had a higher incidence of postoperative complications—most notably pneumonia—and prolonged postoperative recovery. Furthermore, ASA score ≥ 3, moderate preoperative frailty, and low HGS were adverse predictive factors for early postoperative LOI. A simple preoperative risk score incorporating these variables showed moderate accuracy in predicting postoperative LOI.

## Data Availability

The original contributions presented in the study are included in the article/**Supplementary Material**. Further inquiries can be directed to the corresponding author.
