# Supplementary material for: Impact of preoperative handgrip strength and frailty on early postoperative loss of independence in patients with colorectal cancer
Source: Front Oncol. 2026 Jul 1;16:1878444. doi: 10.3389/fonc.2026.1878444 (PMC13368465; doi:10.3389/fonc.2026.1878444)

| **Suppmentary Table 1. The Edmonton Frail Scale** | | | |  |  |
| --- | --- | --- | --- | --- | --- |
| **Frailty domain** | **Item** | **0 point** | **1 point** | **2 points** | **Score** |
| Cognition | Please imagine that this pre-drawn circle is a clock. I would like you to place the numbers in the correct positions then place the hands to indicate a time of ‘ten after eleven’ | No errors | Minor spacing errors | Other errors |  |
| General health status | In the past year, how many times have you been admitted to a hospital? | 0 | 1–2 | ≥2 |  |
|  | In general, how would you describe your health? | ‘Excellent’, ‘Very good’, ‘Good’ | ‘Fair’ | ‘Poor’ |  |
| Functional independence | With how many of the following activities do you require help? (meal preparation, shopping, transportation, telephone, housekeeping, laundry, managing money, taking medications) | 0–1 | 2–4 | 5–8 |  |
| Social support | When you need help, can you count on someone who is willing and able to meet your needs? | Always | Sometimes | Never |  |
| Medication use | Do you use five or more different prescription medications on a regular basis? | No | Yes |  |  |
|  | At times, do you forget to take your prescription medications? | No | Yes |  |  |
| Nutrition | Have you recently lost weight such that your clothing has become looser? | No | Yes |  |  |
| Mood | Do you often feel sad or depressed? | No | Yes |  |  |
| Continence | Do you have a problem with losing control of urine when you don’t want to? | No | Yes |  |  |
| Functional performance | I would like you to sit in this chair with your back and arms resting. Then, when I say ‘GO’, please stand up and walk at a safe and comfortable pace to the mark on the floor (approximately 3 m away), return to the chair and sit down’ | 0–10 s | 11–20 s | One of >20 s patient unwilling, or requires assistance |  |
| Totals | Final score is the sum of column totals |  |  |  |  |

| **Suppmentary Table 2. The Patient Health Questionnaire-9** | | | | |
| --- | --- | --- | --- | --- |
| Name ______________________ Date _________ | | | | |
| Over the last 2 weeks, how often have you been bothered by any of the following problems? | Not at all | Several days | More than half the days | Nearly every day |
| 1. Little interest or pleasure in doing things | 0 | 1 | 2 | 3 |
| 2. Feeling down, depressed, or hopeless | 0 | 1 | 2 | 3 |
| 3. Trouble falling or staying asleep, or sleeping too much | 0 | 1 | 2 | 3 |
| 4. Feeling tired or having little energy | 0 | 1 | 2 | 3 |
| 5. Poor appetite or overeating | 0 | 1 | 2 | 3 |
| 6. Feeling bad about yourself—or that you are a failure or have let yourself or your family down | 0 | 1 | 2 | 3 |
| 7. Trouble concentrating on things, such as reading the newspaper or watching television | 0 | 1 | 2 | 3 |
| 8. Moving or speaking so slowly that other people could have noticed? Or the opposite—being so fidgety or restless that you have been moving around a lot more than usual | 0 | 1 | 2 | 3 |
| 9. Thoughts that you would be better off dead or of hurting yourself in some way | 0 | 1 | 2 | 3 |
| (For office coding: Total Score ____ = ____ + ____ + ____) | | | | |

Supplementary Figure 1. A. Firalty status of patients with colorectal cancer; B. Characteristics of handgrip strength with colorectal cancer patient.


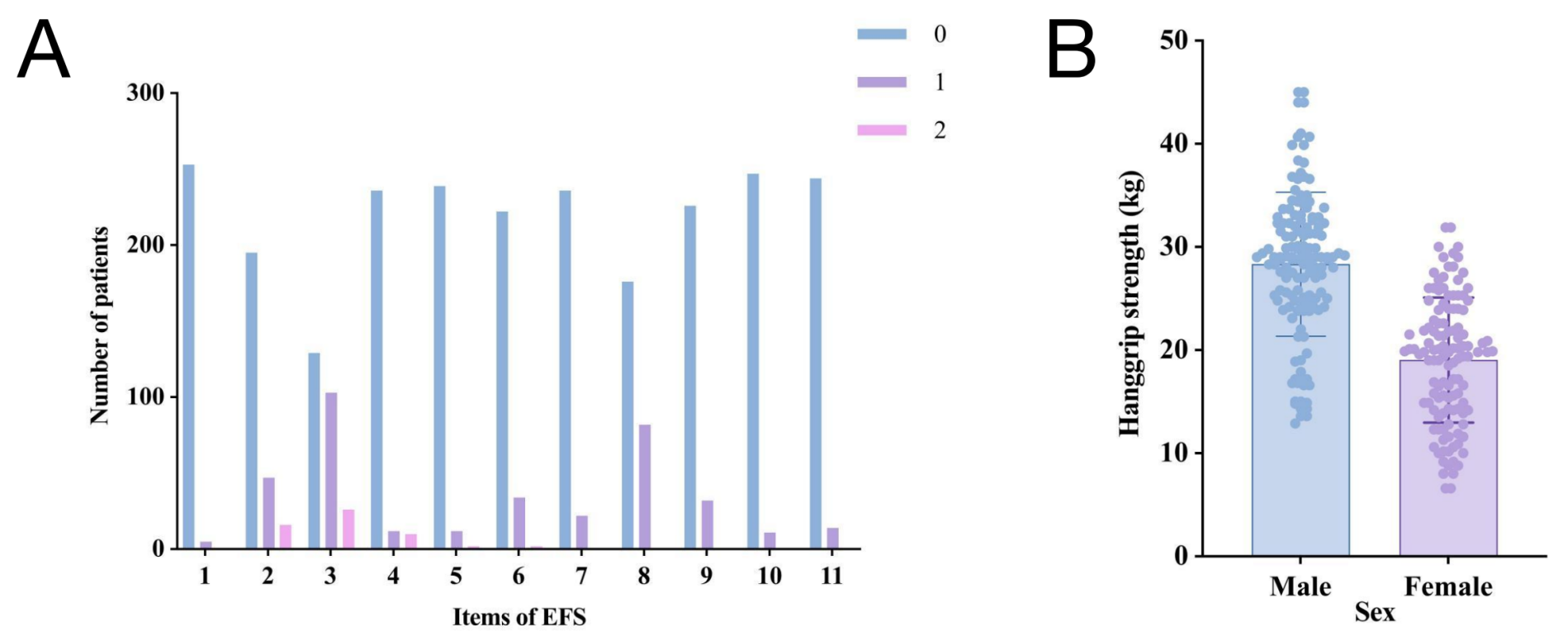


Supplementary Figure 2 Decision curve for prediction model of postoperative LOI.


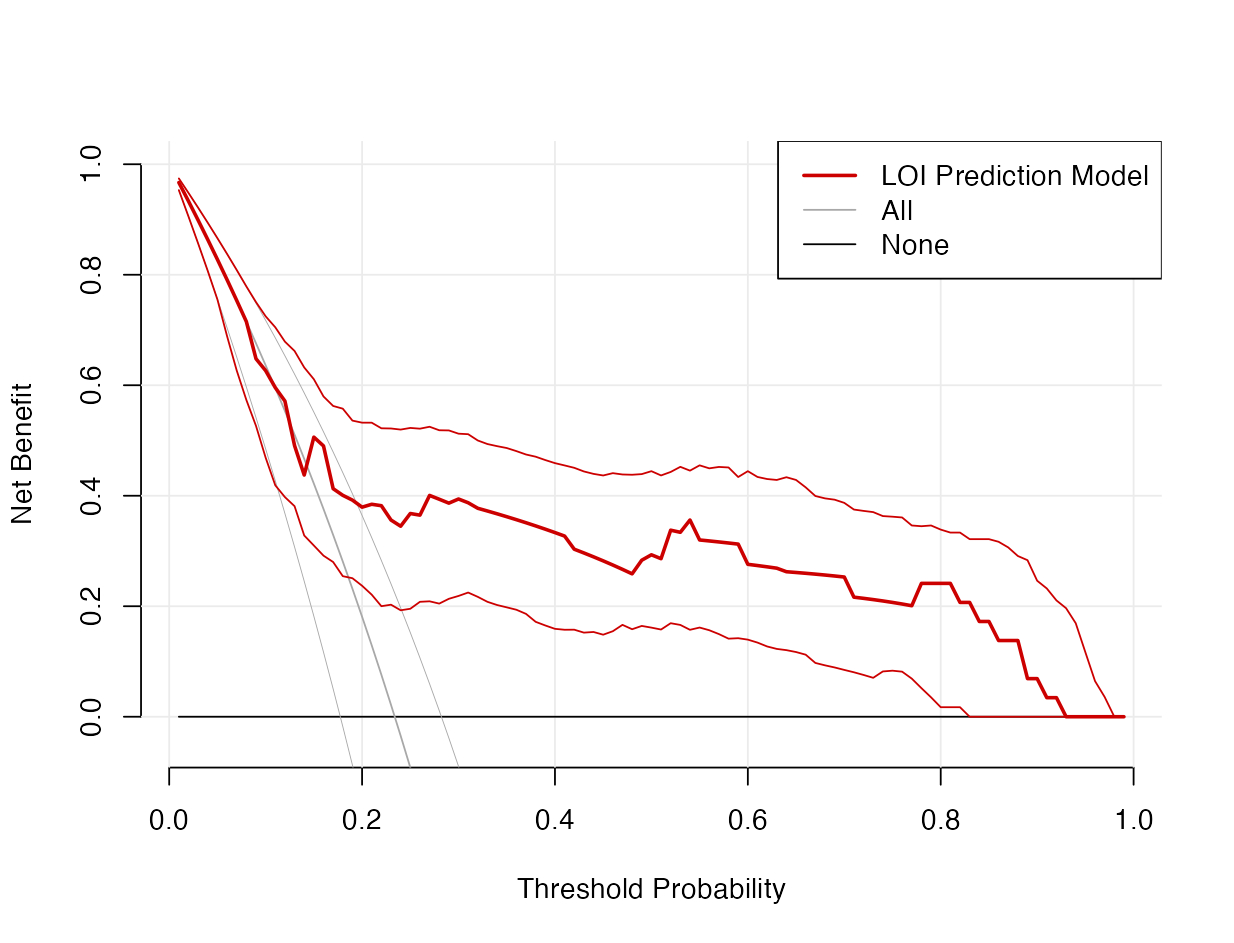

Supplement: Supplementary file 1 [file DataSheet1.docx]
